# The Cognitive Control of Eating and Body Weight: It’s More Than What You “Think”

**DOI:** 10.3389/fpsyg.2019.00062

**Published:** 2019-02-13

**Authors:** Terry L. Davidson, Sabrina Jones, Megan Roy, Richard J. Stevenson

**Affiliations:** ^1^ Center for Behavioral Neuroscience, Department of Psychology, American University, Washington, DC, United States; ^2^ Department of Psychology, Macquarie University, Sydney, NSW, Australia

**Keywords:** obesity, overeating, learning, memory, hippocampus, Western diet, dementia, vicious-cycle model

## Abstract

Over the past decade, a great deal of research has established the importance of cognitive processes in the control of energy intake and body weight. The present paper begins by identifying several of these cognitive processes. We then summarize evidence from human and nonhuman animal models, which shows how excess intake of obesity-promoting Western diet (WD) may have deleterious effects on these cognitive control processes. Findings that these effects may be manifested as early-life deficits in cognitive functioning and may also be associated with the emergence of serious late-life cognitive impairment are described. Consistent with these possibilities, we review evidence, obtained primarily from rodent models, that consuming a WD is associated with the emergence of pathophysiologies in the hippocampus, an important brain substrate for learning, memory, and cognition. The implications of this research for mechanism are discussed within the context of a “vicious-cycle model,” which describes how eating a WD could impair hippocampal function, producing cognitive deficits that promote increased WD intake and body weight gain, which could contribute to further hippocampal dysfunction, cognitive decline, and excess eating and weight gain.

## Introduction

The ability to maintain energy balance and to control one’s body weight involves much more than monitoring bodily energy reserves and the detection of physiological signals that tell when we are hungry or food sated. The decision to obtain and consume food also depends on our knowledge and expectations about food availability, quality (e.g., hedonic, nutritive), the cost and effort required to obtain it, and the potential short-term (e.g., fullness, malaise) and long-term consequences (e.g., on health) of eating it, among other factors. Much of this information resides in memory, and our ability to retrieve the memories of previous experiences with food and eating is an important determinant of our current and future appetitive and consummatory behavior. Furthermore, even when the biological need for food and physical desire to eat is strong we can often resist those urges by avoiding or shifting our attention away from environmental food- and food-related cues that entice us to eat or, failing that, by attempting to suppress the pleasurable thoughts and memories of food and eating that those cues can evoke. Thus, energy intake and body weight regulation involve both metabolic and hormonal control mechanisms and neurocognitive processes involved with memories, expectations, and evaluations about food and the consequences of eating (e.g., Stevenson and Francis, 2017; Yeomans, 2017; Brunstrom and Cheon, 2018; Higgs and Spetter, 2018).

The purpose of this article is to summarize the role of cognitive processes in the control of energy intake and body weight by providing an overview that integrates data and theory from several broad research domains. To achieve this goal, we (1) describe how memory, expectancies, decision making, reward valuation, inhibition, and other cognitive processes can contribute to food intake and body weight regulation; (2) consider evidence from nonhuman and human animals, which indicate that excess food intake and body weight gain negatively impact those processes; (3) review findings, which link this negative impact of diet and obesity to pathophysiologies in brain substrates for learning and memory processes that may contribute to the control of intake; and (4) discuss the possibility that some of these pathophysiological changes may increase the risk of cognitive deficits early in life and for Alzheimer’s disease (AD) and AD-like dementias in late adulthood. The article concludes with the description of a vicious-cycle model that links both obesity and dietary factors to impairments in cognitive processes that are dependent, directly or indirectly, on the functioning of the hippocampus.

## Cognitive Processes Involved with Eating and Body Weight Regulation

### Expectations

#### The Consequences of Eating Can Influence Expectations

The decision to eat and our choice of foods depend in part on the consequences of eating (Higgs, 2016). The consequences of eating refer to more than taste or nutritive impact of foods. The cost of food relative to the value we place on consuming it, considerations associated with the social aspects of eating (e.g., approval/disapproval of others), and cultural constraints about what foods and practices are acceptable are also factors (McClure et al., 2004; Plassmann et al., 2008). Accordingly, our expectations about the outcomes that eating will produce are strongly influenced by memories of our past experiences with eating particular foods and also by the specific contexts (e.g., times, places, situations) in which eating occurred. For example, if we remember enjoying a meal the last time we dined at a particular restaurant or how much we like chocolate ice cream for dessert after dinner, these memories will influence our expectations about the outcomes of future opportunities to dine at that restaurant or eat that dessert (Robinson and Clore, 2002; Kahneman et al., 2004). However, our memory of eating a meal at one restaurant may have limited influence on expectations about enjoying that same meal at a different restaurant. Similarly, expectations about enjoying chocolate ice cream after dinner may be quite different from the expectation about enjoying chocolate ice cream for breakfast. Recall of specific occasions when specific foods were enjoyed (i.e., episodic memories) has been shown to enhance subsequent enjoyment and to increase the likelihood of enjoying and choosing to eat that food again (Robinson et al., 2011b). The fact that such expectations do not appear to generalize broadly to different foods eaten at other times and places (Boutelle and Bouton, 2015; Schyns et al., 2016) indicates that people rely on the memories of specific eating episodes and the contextual retrieval of those memories to predict the hedonic consequences of eating.

#### Expectations Can Influence the Consequences of Eating

While the consequences of eating are important determinants of our expectations, our expectations can also influence the consequences of eating. For example, there is evidence that merely labeling a food as “healthy” reduces the expectation that the food will be liked (Raghunathan et al., 2006). These expectations can be translated to pleasantness ratings following consumption. Schmidt et al. (2017) reported that with healthy participants, the lower pleasantness expectations associated with low-priced wines, as compared to high-priced wines, translated to decreases in experienced pleasantness ratings for lower priced wines, even when those wines were identical to higher priced options. Violations of our expectations can also reduce how much we like a particular food. This was illustrated by Yeomans et al. (2008a) who found that a liking of a pink salmon mousse was much less when it was presented to experimental subjects as sweet ice-cream dessert compared to when the same mousse was presented as a savory appetizer. Similarly, liking by healthy participants of an aroma was found to be much greater when it was believed to be the scent of cheese rather than the smell of body odor (de Araujo et al., 2005). These results show that how much food will be liked depends upon expectations of the person consuming it.

Other studies show that what we remember about our past experiences with a specific food can limit current intake by influencing our expectations about that food’s satiating potential (Irvine et al., 2013) and those expectations can influence the amount of that food we choose to eat (Brunstrom and Shakeshaft, 2009; Wilkinson et al., 2012). Furthermore, manipulations that presumably make it more difficult for rats to form strong expectations about the energy density or caloric consequences of eating a given food have also been reported to promote overeating and weight gain. For example, rats with prior experience consuming both caloric and noncaloric sweeteners appear to be less able to control their intake of calorically sweetened foods, compared to rats that had never consumed artificial sweeteners (Swithers et al., 2010, 2011; Davidson et al., 2011). These findings are consistent with the idea that consuming noncaloric sweeteners augments the intake of sweet high-calorie foods by reducing the expectation that those foods will produce satiety. Previous research with rats also indicated that prior experience consuming a nonnutritive sweetener impaired their ability to regulate their body weight when consuming a sweet, but not a nonsweet, high-fat, and high-calorie maintenance diet. The results suggest that consuming nonnutritive sweeteners promotes increased intake of a sweetened diet and body weight gain by weakening the normally strong predictive relationship between sweet taste and caloric outcomes produced by eating (Davidson et al., 2011). Similarly, in a study with healthy-weight adult humans, Casperson et al. (2017) reported that the reinforcing power of high-sweet snacks was significantly greater relative to salty/savory snacks following intake of nonnutritive sweetened, compared to a sugar-sweetened beverage. This outcome also suggests that exposure to nonnutritive sweeteners selectively augments the ability of sweet tasting, relative to nonsweet foods, to promote appetitive behavior.

There may also be physiological consequences of the mismatch that occurs when sweet tastes are not accompanied by their normal caloric outcomes. For example, in human random control trials, intake of nonnutritive sweeteners has been shown to disrupt glucoregulatory (Lertrit et al., 2018; Romo-Romo et al., 2018) and metabolic (Azad et al., 2017) responses. Thus, weakening the ability to predict the caloric or satiating consequences of a food or fluid may ultimately interfere with metabolic and other responses that contribute to the regulation of energy intake and body weight. A recent study with rodents indicates that this interference might be attributable, in part, to the adverse effects of nonnutritive sweeteners on brain substrates involved with processing this type of information (Erbas et al., 2018).

Viewed from a cognitive perspective, findings that consuming a sweet taste without calories can reduce the expectancy that sweet tastes have caloric consequences is not surprising. Nonetheless, the notion that degrading this expectancy leads to dysregulated energy intake and body weight has been controversial. While there is evidence from studies of human and nonhuman animals that consuming nonnutritive sweeteners similarly can disrupt intake control and metabolic processes (for reviews see Malik et al., 2013; Swithers, 2013; Fowler, 2016; Pearlman et al., 2017), there have also been human randomized control trials and experiments with rats that have failed to obtain such outcomes (e.g., Boakes et al., 2016; for reviews see Miller and Perez, 2014; Tey et al., 2017). Many potentially important parametric and design differences complicate comparison and even interpretation of studies that have reported contradictory outcomes. For example, Boakes et al. (2016) reported that rats previously exposed to saccharin gained more weight when their diet was later supplemented with glucose, compared to rats that received a supplement that contained only saccharin. This could be taken as evidence that saccharin intake produced less weight gain than glucose. However, a second experiment showed that this difference was abolished when saccharin pre-exposure was omitted. One implication of these types of findings is that one’s history consuming nonnutritive sweeteners needs to be accounted for in human or animal studies that attempt to compare the effects of nonnutritive and nutritive sweeteners on energy and body weight regulation. Consistent with previous findings, this pattern of results also suggests that the effects of pre-exposure to saccharin on weight gain may be confined to the regulation of energy consumed in sweet, high-calorie substances (see Davidson et al., 2011; Bissonnette et al., 2017).

### Manipulating the Contents of Memory

Similar to expectations, one’s memory of how much a specific meal was enjoyed can also be manipulated to influence subsequent anticipated enjoyment of that meal. Higgs and her colleagues (Robinson et al., 2011a) asked one group of participants to write down what they enjoyed about a meal that they had just eaten, a manipulation that would presumably strengthen the representation of that meal in memory. A second group wrote down what they enjoyed about a meal they ate a day earlier. To control for the effects of rehearsing the meal without recalling its enjoyable aspects, a third group was asked to recall neutral features of the meal. For each group, actual enjoyment of the meal was the same at the time it was consumed. However, remembered enjoyment of the meal was greater for the group that wrote down what they enjoyed about the meal compared to the group that wrote down the neutral aspects of the meal and the group that had been asked to recall what they enjoyed about a previous meal. Another experiment by the same researchers showed that memory manipulation influenced not only remembered enjoyment of a meal, but also subsequent food choice and amount eaten when participants were offered a subsequent buffet with some of the items that were included in the previous meal.

The amount consumed on one occasion can also be reduced to the extent that one recalls what was consumed at a previous meal, depending on the length of time that separates the two bouts of eating (for reviews see Higgs, 2008, 2016; Higgs et al., 2012a). For example, Higgs (2002) reported that people who were reminded about what they ate at lunch ate less when offered a subsequent snack 3 h later. After 3 h, the effects of the reminders were greatly diminished. One interpretation of this effect is that the memory of the previous meal signals that the likely consequence of eating will be nonrewarding. After three or more hours without food, the memory of a previous meal is less well associated with non-reward and therefore less able to inhibit eating (Davidson et al., 2014b; Higgs, 2016). In support of her overall analysis, Higgs and colleagues also found that enhancing the encoding of memories of a previous meal by requiring extra chewing reduced subsequent snack intake (Higgs and Jones, 2013). It should be noted that the act of chewing alone, even in the absence of calories, taste, or odors, can suppress appetitive behavior (Ikeda et al., 2018). In contrast, interfering with the encoding of a previous meal by exposing participants to distracting activities (e.g., watching TV, playing a video game) while eating increases subsequent snack intake (Higgs and Woodward, 2009; Mittal et al., 2011; Oldham-Cooper et al., 2011; Higgs, 2015). The effect of encoding interference may be sex specific. Woman snacked more during periods of distraction, but men who were distracted while snacking increased their intake at a later meal (Francis et al., 2017). More research should be done to examine how sex interacts with distraction to alter memory encoding and appetitive behavior.

Support for the idea that memory of recent eating can inhibit subsequent intake also comes from data that show better episodic memory recall is associated with an increased cognitive restraint and a decreased uncontrolled eating, making episodic memory a reliable predictor of appetitive behavior (Martin et al., 2018). Furthermore, amnesic patients who are unable to recall recent eating will consume multiple full meals in quick succession (Hebben et al., 1985; Higgs et al., 2008).

### Perception and Memory

The perception of how much we have eaten and memories about the effects of eating on our level of hunger and satiety can combine to exert a strong influence on intake. For instance, in a study by Brunstrom et al. (2012) subjects ate from bowls that contained either 500 or 300 ml of soup. Unbeknownst to the subjects, the bowls were secretly filled or emptied by the experimenters while the subjects ate. Thus, how much soup they actually ate was sometimes the same (ate 500 ml, saw 500 ml; ate 300 ml, saw 300 ml), sometimes greater than (ate 500 ml, saw 300 ml), and sometimes less than (ate 300 ml, saw 500 ml) the portion size they thought they had consumed. When asked to evaluate their level of hunger 2–3 h after eating, the subjects who believed that they ate 500 ml of soup rated themselves as less hungry than did the subjects who believed they ate 300 ml, regardless of the actual amount of soup they had eaten. The results provide evidence that one’s perceptions of how much they have eaten and their memories of past experiences eating given amounts of food provide information that people use to determine their level of satiation.

Perceptions can also have impact independent of the amount of food that is thought to have been consumed. Experiments by Robinson et al. (2016) showed that the one’s definition of a normal-size meal could be increased by presentation of visual images that depicted meals of different sizes. Specifically, viewing an image of a larger-than-normal meal increases the size of a meal that is perceived to be normal. However, the amount of actual food consumption did not change for participants whose perceptions of normal meal size had been altered upward. Additional research on the relationship between perception of normal meal size and actual consumption is warranted.

### Attentional Biases Toward Food Cues

Another way memory may contribute to the control of appetite and eating is by guiding attention toward or away from food-related cues in the environment. That is, individuals can inhibit particular appetitive behaviors by directing their attention away from food-related cues that can evoke those responses. Similarly, thinking about food may increase the likelihood that a person will notice and respond to food-related cues in their surroundings. Support for this general principle comes from studies which show that forming a mental image of an object (to activate the image in working memory) increases the amount of attention given to similar objects that were presented in a visual search task (e.g., Soto et al., 2005). A study by Higgs et al. (2012b) confirmed that attentional bias, defined here as heightened attention toward food cues (e.g., Hollitt et al., 2010), could also be produced simply by asking people to memorize a picture of food. Furthermore, “unsuccessful dieters,” defined as people with high restraint and high disinhibition, showed a higher tendency to attend to food-related stimuli when holding food cues in working memory compared to successful dieters who were high in restraint but low in disinhibition (Higgs et al., 2015). These findings indicate that when the ability to control thoughts about food is weakened, reactivity to food cues and external eating is likely to increase, particularly in already disinhibited eaters. However, Whitelock et al. (2018) were unable to obtain evidence that manipulating attention to food cues during intake was associated with changes in subsequent intake. Noting that the strength of that memory is the main determinant of subsequent intake (see “Manipulating the Contents of Memory” above), these researchers suggested that their negative findings may reflect that their attention manipulations (e.g., verbal instructions to pay attention to the sensory properties of a meal) were ineffective at enhancing the memory of the meal.

The evidence pertaining to whether attentional biases to food cues cause weight gain in humans is also mixed. Such an effect has been reported for “external eaters” for which food-related stimuli can evoke eating responses, largely independent of hunger or satiety state (Tapper et al., 2008; Brignell et al., 2009; Nijs et al., 2009; Hepworth et al., 2010; Hou et al., 2011). However, restrained eaters and people suffering from anorexia nervosa also exhibit attentional biases to food cues despite the attempts of these individuals to exert strong control over eating (see Brooks et al. (2011). Results of a study by Hollitt et al. (2010), illustrates some of the complexity involved with the relationship between restrained eating styles and attention. They found that restrained eaters exhibit increased vigilance that enables them to quickly detect food cues, but once detected the capacity of food cues to hold the attention of restrained eaters does not differentiate them from individuals that exhibit less restrained eating styles. One implication of these findings is that the relationship observed between restrained eating and attention is likely to depend on when and how attention to food cues is measured.

The extent to which attentional bias contributes to overeating appears to depend on the types of foods that are the targets of the bias. Calitri et al. (2010) reported that people with attentional biases to words associated with unhealthy foods exhibited increased BMI during a 12-week weight loss intervention, an attentional bias to healthy food words was associated with lower BMI. Furthermore, attentional biases to high-calorie food cues have also been associated with poor dieting success by restrained eaters who engage in effortful food intake control (Meule et al., 2012). Thus, it appears that attentional biases could either help or hinder efforts to control food intake, depending on the types of foods that are attended to. Recent experiments have attempted to capitalize on the possibility that increasing a person’s attention to healthy foods, (Kakoschke et al., 2014) and decreasing their attention to unhealthy foods, might promote healthy eating (Hardman et al., 2013). While the effectiveness of this approach at changing eating habits remains to be established, it has encouraged the view that one way to combat overeating and obesity is to engage in more “mindful” eating (see Higgs et al., 2012a; Bazzaz et al., 2017).

There is also evidence that, at least for external eaters, these attentional biases to food-related cues are a consequence of a broader impairment in attention. Higher scores on scales of external eating are associated with greater difficulty in the control of cognitive processes leading to impaired task-shifting, greater distractibility, reduced ability to suppress unwanted thoughts (Ebneter et al., 2012). While further research is needed, poor control of food intake by people that exhibit attentional bias to food cues might be one manifestation of a more general attentional deficit.

### Inhibition

Sometimes, eating appears to be a reflexive, automatic, response that can be evoked, without expectations or the operation of any conscious decision-making process, by the mere sight, smell, or even the thought of a favored food (Jones et al., 2018a). This type of impulsive behavior can be opposed by dietary restraint, which involves reliance on higher-level cognitive processes to counter the power of palatable food to evoke eating (Herman and Mack, 1975; Polivy and Herman, 1985). One function of dietary restraint is maintaining a balance between conflicting short-term and long-term goals (Rangel, 2013). A short-term goal would be obtaining the pleasurable outcome associated with consuming a favorite sweet. A longer-term goal might be to maintain health or current body weight. Poor inhibitory control of behavior, often described as “impulsivity” or poor “self-regulation,” would produce an imbalance favoring pursuit of the short term over the longer-term goals, leading to overconsumption of palatable foods (Carr et al., 2011; Hall, 2012; Price et al., 2015; Bazzaz et al., 2017; Dohle et al., 2018). Thus, in terms of cognitive control, to maintain a healthy diet, prevent weight gain, lose weight or prevent weight regain, one must strive to inhibit thoughts about tasty, high-calorie foods and beverages that can elicit eating.

Support for the hypothesis that overeating could be a consequence of a failure to inhibit retrieval of thoughts and memories related to food comes from a variety of studies using cognitive and behavioral measures. For example, manipulations that have been shown to increase memory intrusions in nonhuman animals are accompanied by increases in food-cue reactivity, food intake, and body weight (for reviews see Kanoski and Davidson, 2011; Kanoski, 2012). In humans, it also appears that individuals who exhibit poor control over their eating behavior can be differentiated from people with strong control based on their cognitive inhibitory function. For instance, children with better self-regulatory skills maintain better health and smaller increases in BMI across childhood and adolescence (Bub et al., 2016). Furthermore, compared to nonobese people, obese restrained eaters (who monitor their food intake carefully) are more susceptible to memory intrusions, including intrusions of food memories (Laessle et al., 1989; Soetens and Braetchara, 2006) that are characteristic of food cravings (see Stevenson and Francis, 2017). Similarly, memory intrusions are more likely to be experienced by restrained eaters that are also susceptible to eating evoked by environmental food cues (Soetens et al., 2008a; but see Soetens and Braet, 2007). These findings suggest that people who lack control over their food intake have cognitive inhibitory deficits that reduce their ability to suppress thinking about food and food-related environmental stimuli. In contrast, strong inhibitory control should help to reduce intake to the extent that it helps people to ignore powerful environmental food cues that seem to entice overeating (see Appelhans, 2009). Inhibition of attention to food-related cues in the environment and inhibition of memories of past positive experiences with food and eating likely interact to reduce energy intake (e.g., Depue et al., 2010).

Unfortunately, attempts to ban unwanted thoughts can be counterproductive, with the frequency of the unwanted thoughts increasing rather than decreasing (Abramowitz et al., 2001). This means that trying to control one’s thoughts about food could lead to an undesirable increase in food-related thoughts and memories. In fact, restrained eaters that exhibit both a strong preoccupation with food and a weak control of their food intake often paradoxically report an increased frequency of food-related thoughts after attempting to ban those thoughts from consciousness (Soetens and Braet, 2006). Therefore, thought suppression strategies can result in overeating for some people (Soetens et al., 2008b; Erskine and Georgiou, 2010).

## Effects of Diet/Obesity on the Cognitive Controls Of Behavior

### Cognitive Control of Food Intake

If the control of eating depends, in part, on cognitive functioning, one would expect that people who overeat and become obese would exhibit impaired cognitive control. In particular, weaknesses in the ability to suppress the retrieval of memories or thoughts of food, or to shift attention away from those memories, thoughts or food-related environmental cues should be associated with a higher incidence of impulsive eating behavior and excess body weight.

Consistent with this possibility, increased food intake has been associated with impaired inhibitory control in several studies using neurocognitive tests (Guerrieri et al., 2008; Yeomans et al., 2008b; Jansen et al., 2009; Sellaro and Colzato, 2017; Chojnacki et al., 2018). For instance, in a go/no-go task, response inhibition by obese individuals was inversely related to body weight and adiposity (Kamijo et al., 2012; Wirt et al., 2014). Other research showed that the amount of weight loss exhibited by obese individuals during a treatment program was positively related to the level of behavioral control they exhibited at baseline (Nederkoorn et al., 2006a, 2007, 2010). Furthermore, following weight loss, lower neural activation in brain frontal areas underlying inhibitory control (e.g., superior, middle, and inferior frontal gyri; medial prefrontal cortex) predicted weight regain, whereas increased activation was associated with maintenance of lower body weight (McCaffery et al., 2009; Batterink et al., 2010; Kishinevsky et al., 2012).

Obese people appear to be at greater risk of losing control over their own actions when their actions earn rewards. For example, when required to make a response that earns an immediate small reward or a response that earns a delayed but larger reward (i.e., a delayed discounting task), obese people are less likely than normal-weight people to choose the response that requires them to wait for the larger, delayed reward (Sigal and Adler, 1976; Johnson et al., 1978; Bonato and Boland, 1983; Weller et al., 2008). However, early studies reported mixed results in delayed gratification tasks that compared obese with nonobese children (e.g., Bourget and White, 1984) and there is some evidence that impaired delay of gratification in obese adults emerges only at later phases of testing (Nederkoorn et al., 2006b).

Compared to nonobese individuals, obese people have been reported to exhibit more persistent responding, even as the probability of receiving a reward gets smaller when their rate of responding gets higher (Nederkoorn et al., 2006a; Verbeken et al., 2009). Additionally, preferential choice for smaller, more immediate, food rewards is negatively associated with success in obesity treatment programs (Best et al., 2012). The results of human correlational studies have been supported by animal research findings that indicate the introduction of high-fat, high-sugar diets is *induces impulsive choice behavior* (Steele et al., 2017). It also appears that the positive relationship between impulsivity and obesity is strongest in obese individuals that also exhibit binge eating (Giel et al., 2017). Overall, the results suggest that obese people are more likely to respond impulsively for food rewards and less likely to stop responding even as the value of those rewards diminishes. These effects may be a result of the consumption of high-energy diets.

Evidence implicating poor inhibitory control in obesity also comes from findings that experimental manipulations that weaken response inhibition also increase food intake and vice versa. In one such study, Guerrieri et al. (2012) reported subjects that performed a stop signal task ate more during a subsequent intake test, if the version of the task they were given prioritized “go” trials relative to “stop” trials. Other studies have shown that giving individuals “inhibitory control training” (ICT) which enables them to practice inhibiting their responses to cues for a particular food can be successful at decreasing intake of that food (Houben and Jansen, 2011). The effectiveness of ICT has been supported by the results of meta-analyses. Allom et al. (2016) analyzed the findings of 19 studies and concluded that training on a go/no-go training significantly improved health behaviors, at least on a short-term basis. A second meta-analysis (Jones et al., 2016) confirmed and extended the results of Allom et al. (2016) primarily by providing evidence that an associative mechanism was responsible for the positive effects of ICT. Overall, these results indicate that inhibitory control and control of food intake are closely related and that inhibitory deficits may precede weight gain and obesity (Stevenson, 2017).

### General Impairments in Cognitive Functioning

In addition to research on food-focused cognitive controls of intake, there is also evidence that overeating and excess body weight may be a result of more general impairments in cognitive functioning. For example, reduced physical activity, increased dietary intake, and other lifestyle factors related to obesity have been found to co-occur with weakened episodic memory (Loprinzi and Frith, 2018). High BMI and disinhibition scores are also predictive of slower completion times on a standard color-word Stroop task (Maayan et al., 2011; but see Phelan et al., 2011). Furthermore, childhood inhibitory control has been linked to the development of weight gain over time. For example, in a prospective study, Nederkoorn et al. (2007) reported that poor inhibitory control in children was associated with higher body weights 6 and 12 months later, with the most impulsive children exhibiting the most weight gain. In addition, Schlam et al. (2013) reported that poorer inhibitory control at age 4, as indicated by performance on a delay of gratification task, was associated with higher BMI 30 years later. One study found that children with poorer inhibitory control had significantly higher body weights 6 and 12 months later, with the greatest weight gain occurring in the most impulsive children (26). Indeed, a recent longitudinal study found that poor impulse control at age 4 was associated with a higher BMI 30 years later (27). In addition, Stangl and Thuret (2009) found that while obese children generally exhibited poorer inhibitory control than lean children, this was exacerbated even further in a subsample of obese children who exhibited binge eating behavior (see also Crews and Masliah, 2010). While preliminary, these findings suggest that, for some people, loss of control over food intake may be symptomatic of a more general impairment in cognitive functioning.

## Diet, Obesity, and Cognitive Decline

### Cognitive Impairments Earlier in Life

Being overweight or obese is not only linked to cognitive deficits in late adulthood. Reduced performance on measures of cognitive functioning was observed throughout the stages of life. When interpreting the results of studies that provide evidence of obesity-related impairments on tests of cognitive functioning, the possibility that test performance is influenced by nonspecific factors (e.g., motivational, arousal) that could have global impact on cognitive functioning must be kept in mind. This concern is reduced in the studies which reported that obesity-related deficits were selective for some cognitive functions while others were spared, even though both the impaired and spared functions were presumably subject to the same nonspecific influences. For example, in preadolescent children, BMI and body fat were inversely correlated with response inhibition and academic achievement (Kamijo et al., 2012, 2014; Blanco-Gomez et al., 2015), but this relationship was not observed in cognitive tasks that required smaller amounts of inhibitory control. In another study that tested the set shifting ability of obese and lean 12-year-old boys, reduced attention endurance and increased perseverative errors were associated with obesity, but performance on tests of verbal fluency and digit span memory were not (Cserjesi et al., 2007). Similarly, Verdejo-Garcia et al. (2010) reported that compared to adolescents (13–16 years old) normal-weight obese adolescents performed worse on measures of cognitive flexibility, decision making, and inhibition, but not on tests of planning, reasoning, and working memory. Deficits appear to continue into young adulthood, as higher BMI has been associated with poor performance on episodic memory tasks in 18–35-year olds (Cheke et al., 2016; but see Cole and Pauly-Takacs, 2017). In this study, BMI was unrelated to encoding time, indicating that overweight participants did not devote less effort to memorizing the information on which they were tested.

In addition to the performance deficits noted above, obesity has also been linked to childhood impairments in specific types of memory processes. For example, in children 7–9 years of age, both body adiposity (Khan et al., 2015) and self-reported fat intake (Baym et al., 2014) were found to be negatively correlated with relational memory (i.e., the ability to encode and remember relations, e.g., spatial, temporal, associative) among items in a visual scene, but were uncorrelated with memory for individual items. Because relational memory is also thought to rely on processes that are distinct from those that underlie executive function, these results suggest that obesity, and diets associated with obesity, may have an even broader impact on childhood cognitive and neural functioning than recognized previously.

Just as obesity is able to predict performance impairments on cognitive tasks, it seems that such impairments can also predict obesity. Nelson et al. (2017) ran a longitudinal study with 212 children from preschool to grade 4 examining the relationship between executive functioning and weight status. Findings from this study revealed impairments in executive control that emerge as early as preschool are associated with a greater risk for overweight or obesity status in elementary school. These results indicate impairments to cognitive control may precede and promote weight gain.

### Late-Life Cognitive Decline

Worldwide in 2018, an estimated 50 million people were victims of dementia with projected incidence nearly tripling by 2050 (World Alzheimer Report 2018, 2018). As a clinical syndrome, the criterion for a diagnosis of dementia is progressive decline in two or more cognitive domains, including memory, language, executive function, affect, and personality, which makes one unable to perform the basic activities of daily life (Weller and Budson, 2018). A recent meta-analysis estimated the relative strengths of several health and lifestyle factors that have been deemed to influence the risk of cognitive decline (Baumgart et al., 2015). Mid-life obesity and two of its comorbidities, mid-life hypertension and diabetes, were among the strongest risk factors, whereas Mediterranean diet (relatively low in saturated fat and sugar) and physical activity were associated with decreased risk (also see Whitmer et al., 2008; Hassing et al., 2009; Alford et al., 2018). Increased incidence of serious late-life dementia has also been linked to high levels of central adiposity (i.e., abdominal body fat) during middle age (Profenno et al., 2010). Furthermore, association between Type 2 diabetes and AD is strong enough that some investigators have referred to AD as “Type-3” (Kandimalla et al., 2017) or “brain” diabetes (Leszek et al., 2017).

## Western Diet (WD) and Cognition

### The Western Dietary Pattern

The Western dietary pattern is considered a significant contributor to the increases in body weight, adiposity, and metabolic disease (e.g., Type II diabetes) that have been taking place over the past 40 years in Western and westernized societies (Medina-Remon et al., 2018). The 2015 Dietary Guidelines Advisory Committee Scientific Report concluded that the overall US population consumes lower than recommended amounts of vegetables, fruits, whole grains, and dairy and higher than recommended amounts of refined grains, added sugars, salt, and saturated fats derived from red meats. This pattern of low-quality diet is prevalent regardless where the diets are prepared or eaten (Millen et al., 2016). The eating habits of the US population described by this report define the Western dietary pattern (Cordain et al., 2005). The Western dietary pattern is deemed “unhealthy” for humans based on a variety of epidemiological findings linking it not only to obesity and metabolic disease, but also to increased risk of stroke (Sherzai et al., 2012), colorectal cancer (Yusof et al., 2012), kidney disease (Hariharan et al., 2015), and immune system dysfunction (Myles, 2014).

### WD and Cognitive Functioning

Western diet intake is also associated with both mild and more severe forms of cognitive impairment (e.g., Yeomans, 2017; Kivipelto et al., 2018). For example, the diets of Polish patients diagnosed with AD included high amounts of meat, eggs, high-fat dairy, butter, and sugar, whereas non-demented, age-matched people tended to consume more vegetables and grains. In another epidemiological study, Eskelinen et al. (2008) found that people who consume a diet containing a high level of saturated fat at mid-life were at greater risk for mild cognitive impairment in late adulthood compared to people that consumed less saturated fat at mid-life.

Experimental studies also provide evidence that links WD and obesity with cognitive impairments. Cohen et al. (2011) found that obese older adults 50–69 years of age exhibited more perseverative errors, worse attention and ability to concentrate, and slower processing speed than their lean, aged-matched counterparts. The self-reported diet of the lean group also included more produce, fish, nuts, and whole grain foods, and less meat, fried food, fast food, alcoholic beverages than that of the obese group. Similarly, college students that reported high preference for junk food, such as potato chips, nachos, and candy bars, and low preference for fruits and vegetables exhibited weaker attentional and motor response control compared to students that did not express this pattern of preferences (Jasinska et al., 2012). In addition, Francis and Stevenson (2011) found that undergraduate university students who self-reported consuming higher levels of WD exhibited poorer performance on a hippocampal-dependent logical memory task, but not on a hippocampal-independent control task, compared to students that reported a less adherence to the WD. Finally, Riggs et al. (2010) found that for 7- to 9-year-old children, greater self-reported intake of snack foods and less consumption of fruits and vegetables were associated with poorer performance on an index composed of measures on inhibitory and emotional control, along with working memory and planning and organizational aptitude.

Memory impairments have been observed following very short periods of WD exposure. Attuquayefio et al. (2017) found impairments on multiple memory tasks (e.g., Hopkins Verbal Learning Task-Revised, Logical Memory) following 4 days of eating breakfasts high in saturated fat and added sugar, as compared to a healthier breakfast. Similarly, in animal research, short-term exposure to high-energy diets, ranging from 10 to 14 days, has been found to impair memory and cognitive function (Kanoski and Davidson, 2010; Beilharz et al., 2014, 2016; Jones et al., 2018c). It appears that at least some rapidly induced impairments (e.g., following 15 days on WD) can be reversed after WD intake is suspended (Tran and Westbrook, 2017; McLean et al., 2018). However, a study with mice examined reversibility of the effects of 15-week WD exposure and found that while the adverse effects of WD on bodyweight and metabolic function were reversed, little recovery of deficits in hippocampal-dependent water-maze and contextual memory performance was observed 60 weeks after suspension of WD (Wang et al., 2015).

## Physiological Mechanisms Underlying the Adverse Effects of the Western Dietary Pattern on Cognitive Function

### The Hippocampus, Memory, and Cognition

As discussed above, the Western dietary pattern can have deleterious effects on food intake, body weight, and cognitive functioning. The findings also indicate that excess food intake and body weight at a younger age may be related to the development of severe impairments in cognitive functioning later in life. While early cognitive impairments and signs of pathophysiology may be relatively subtle, both may be predictors of the appearance of increasingly serious dysfunction later in life. Several researchers have attempted to map the emergence of cognitive dysfunction from subtle deficits to mild cognitive impairment, to full-blown dementia, across the lifespan. It appears that the first signs of cognitive disease can emerge 50 or more years before the diagnosis of serious cognitive dysfunction (Smith, 2002). The earliest markers for disease have been identified in the hippocampal formation (Khan et al., 2014), which comprises the CA1-CA3 cell fields of the hippocampus proper, along with the dentate gyrus, the subicular complex, and the entorhinal cortex (Amaral and Witter, 1989; Schultz and Engelhardt, 2014). With the passage of time, there is a spread of pathophysiology into the other limbic and cortical areas that have connections with the hippocampus (Didic et al., 2011; Kiernan, 2012).

Both humans and rats that have experienced damage to the hippocampus, and/or the larger hippocampal formation, exhibit deficits in memory and cognition. Indeed, much of the structure and many functions of the hippocampus appear to be conserved across human and rat species (Clark and Squire, 2013). In humans, functional deficits include dense anterograde amnesia (i.e., loss of the ability to remember new information) and impairments in the ability to remember spatial relationships such as those used for navigation to and from different places in the environment (Aggleton, 2014; Esfahani-Bayerl et al., 2016). In other human studies, functional magnetic resonance imaging (fMRI) provided evidence for hippocampal involvement in withholding of previously rewarded responses (Kaag et al., 2016; Kruse et al., 2016) and in the inhibition of intrusive memories (Anderson et al., 2016). Both spatial and nonspatial memory processes are also impaired in rats as a result of selective hippocampal damage. For example, rats that have sustained highly selective hippocampal lesions are impaired in spatial memory tests that require retrieval of location of a hidden platform in a water maze (e.g., Morris et al., 1990), the location of arms in a radial maze that contain food rewards (e.g., Jarrard et al., 2012), and on other location-based foraging tasks (Evans et al., 2018).

Relative to intact controls, rats with hippocampal damage are also impaired solving nonspatial conditional discrimination problems in which discrete or diffuse contextual cues signal when other events will be followed by reinforcement (Holland et al., 1999; Riaz et al., 2017). In contrast, performance of the same rats is not impaired on similar hippocampal-independent tasks that do not require the use of spatial memories (e.g., locating a visible platform in a water maze) or that require learning only simple relationships involving nonspatial stimuli (e.g., learning to discriminate between one rewarded cue and a different nonrewarded cue; Daum et al., 1992; Gerlai, 2001).

### The Hippocampus, Energy Intake, and Body Weight Regulation

The hippocampus also has a role in eating and body weight regulation. Humans with medial temporal lobe damage (an area which includes the hippocampus) have difficulty detecting or utilizing the information provided by their internal cues (Berriman et al., 2016), including interoceptive stimuli corresponding to hunger and satiety (Hebben et al., 1985; Dudley and Stevenson, 2016). Rats with hippocampal damage or with temporary inactivation of the hippocampus induced by anesthesia also exhibit signs of impaired energy regulation in the form of increased appetitive behavior, eating, and body weight gain relative to intact controls (Clifton et al., 1998; Davidson et al., 2009). They also exhibit shorter intervals between meals (Henderson et al., 2013) and impaired ability to discriminate between interoceptive cues produced by different levels of food deprivation (Davidson et al., 2010). Recent findings also show that the inhibition of food intake depends, in part, on activation of a neural pathway between the hippocampus and the lateral hypothalamus, an area of the brain that has been implicated in the production of satiety (Sweeney and Yang, 2015).

There are many similarities between the effects of hippocampal damage on food intake and cognitive functioning and those of WD. Rats fed ad libitum WD also show performance deficits, compared to chow-fed controls, in the water maze (Molteni et al., 2002), Y-maze (Hargrave et al., 2016), radial-arm maze, (Kanoski and Davidson, 2010), object place recognition (Beilharz et al., 2014), conditional discrimination problems (Davidson et al., 2012), and other hippocampal-dependent tasks (e.g., Boitard et al., 2014; Cordner and Tamashiro, 2015; Tran and Westbrook, 2015). In addition, when the performance of the WD-fed rats was assessed in hippocampal-independent tasks, no impairments were observed (Tran and Westbrook 2015 and Davidson et al. 2012). Findings that performance is selectively impaired in hippocampal-dependent problems make it difficult to claim that these impairments are a consequence of changes in reward, motivation, arousal, behavioral competency, or other global deficits because these types of changes would be expected to impact performance on both types of problems. Rather, the results indicate that WD intake produced a selective impairment in hippocampal function. In addition, like hippocampal damage, WD intake has been reported to increase food intake, body weight, appetitive responding to external food cues, and to impair discrimination of internal states (Newby et al., 2003; Francis and Stevenson, 2011; Brannigan et al., 2015; Attuquayefio et al., 2016; Sample et al., 2016, 2018).

### The Effects of WD and Obesity on the Hippocampus

WD intake has disruptive effects on hippocampal-dependent cognitive processes, on hippocampal structural and functional integrity and may be involved with the pathogenesis of AD. Research from several laboratories shows that rodents maintained on WD exhibit reduced neurogenesis and increased inflammation in the hippocampus, along with a selective weakening of the hippocampal blood-brain barrier (BBB; Stangl and Thuret, 2009; Kanoski et al., 2010; Grayson et al., 2014). Reduced hippocampal volume has also been observed in obese children (Mestre et al., 2017) and overweight and obese adults (Cherbuin et al., 2015). Independent research also indicates that reduced hippocampal neurogenesis (e.g., Crews and Masliah, 2010; Lazarov and Marr, 2010), reduced hippocampal volume (e.g., Peng et al., 2015), increased brain inflammation (Cuello et al., 2010; Zhao et al., 2011), and decreased BBB function (Ryu and McLarnon, 2009; Coisne and Engelhardt, 2011; Zlokovic, 2011; Montagne et al., 2015) are also important components of the pathogenesis of AD in humans (e.g., Pimplikar et al., 2010; Griffin, 2011).

#### The Blood-Brain Barrier

The BBB maintains brain health by regulating the transport of nutrients and preventing access to the brain of toxicants that are carried in the blood (Zhao et al., 2015). When the structural integrity of the BBB is compromised, disruption of transporter functions can reduce nutrient supply and increased permeability can allow circulating neurotoxic substances to leak into the brain (Zheng et al., 2003; Liebner et al., 2018). These signs of pathology are often most pronounced in the hippocampal formation (Hargrave et al., 2016), which may make the hippocampus selectively vulnerable to a number of different insults (Williamson and Bilbo, 2013).

Obese rats maintained on WD, or similar high-energy diets, exhibit selective permeability of the hippocampal BBB (Freeman et al., 2014). This has been demonstrated by findings showing that leakage of sodium fluorescein (a small molecule dye that cannot readily cross an intact BBB) is increased for obese rats that had been maintained on WD relative to leaner, chow-fed controls. (Kanoski et al., 2010; Davidson et al., 2012, 2013). In addition, WD-induced obesity is also accompanied by reductions in the expression of tight junction proteins that control entry of substances in the gaps between endothelial cells in the BBB (Kanoski et al., 2010). Rat models of Type II diabetes have reported similar disruptions in BBB function (Yoo et al., 2016; Takechi et al., 2017). Changes in BBB permeability have been observed after 90 days of WD maintenance (Hargrave et al., 2016), though one study using somewhat older rats detected WD-induced increases in permeability after just 28 days of diet exposure (Davidson et al., 2012). In these experiments, BBB permeability was not increased in the striatum or prefrontal cortex of WD-fed rats, relative to rats fed chow.

In two studies (Davidson et al., 2012, 2013), only WD-fed rats that gained the most weight and body fat (diet-induced obese (DIO) rats) exhibited significantly increased hippocampal BBB permeability compared to rats fed standard low-fat, low-sugar chow. Increased BBB permeability was not observed in diet-resistant (DR) rats for which WD intake did not increase body weight, relative to chow-fed controls. Interestingly, the DIO, but not DR rats, also showed impaired performance on a discrimination problem that depends on the hippocampus. Neither of these groups was impaired on a hippocampal-independent discrimination. These results show that selective increases in BBB permeability and selective impairment in hippocampal-dependent cognitive functioning co-occur in rats that become obese when maintained on a WD.

#### Glucose Transport

It seems unlikely that increased BBB permeability underlies all impairments in hippocampal-dependent learning and memory functions. Several studies have shown that maintenance on WD for 10 days or less can disrupt performance on these types of tasks (Murray et al., 2009; Kanoski and Davidson, 2010; Jones et al., 2018b). Changes in BBB permeability appear to require a longer period of exposure to ad libitum WD (Hargrave et al., 2016). However, recent research suggests that such early cognitive deficits might be explained by disruption of glucose transport across the BBB. The brain depends on glucose as its primary source of energy. Glucose, the primary energy source for the brain, relies on transporters, predominantly glucose transporter 1 (GLUT-1), to cross the BBB. (Takata et al., 1990). Following 3–7 days of maintenance on a high-fat diet, Jais et al. (2016) found that GLUT-1 expression at the BBB and glucose uptake in the brains of mice were significantly reduced. This disruption in GLUT-1 recovered after 30 days of prolonged diet exposure. A follow-up study showed that a high-fat diet induced a decrease of GLUT1 in humans, an effect that was associated with performance deficits on a hippocampal-dependent auditory verbal learning task (Schuler et al., 2018). A study with rats by Hargrave et al. (2015) confirmed that the expression of GLUT-1 was reduced at the hippocampal BBB following 10 days of ad libitum WD and showed that this reduction in GLUT-1 expression was also observed, although at somewhat smaller magnitudes following both 40 and 90 days of WD exposure. One implication of these results is that WD intake may disrupt hippocampal functions by first decreasing glucose transport and later by increasing permeability at the hippocampal BBB (Winkler et al., 2015).

#### Inflammation

Microglia act in the brain like first responders to deviations from normal homeostasis that could result from the entry of pathogens or other types of insults (Beilharz et al., 2015). After migrating to the site of the insult, microglia activate several immune system functions such as the release of proinflammatory cytokines, which help to isolate the disruptive event, along with neurotrophic factors that promote neurogenesis and neural repair (Dheen et al., 2007). Thus, inflammation is a sign of underlying brain pathophysiology. Diets high in fat and sugar have been reported to induce inflammation in the hippocampus (Sobesky et al., 2014; Boitard et al., 2014; Guillemot-Legris and Muccioli, 2017). Increased expression of proinflammatory cytokines has been observed in the hippocampus of rats given either a cafeteria diet or a diet of standard chow along with a 10% sucrose solution (Beilharz Maniam and Morris 2014). Level of inflammation is also inversely related to performance on a hippocampal-dependent spatial memory problem (Beilharz et al., 2014). Deficits in hippocampal-dependent memory, accompanied by inflammation, have also been reported following 1 month on a chow diet supplemented with high-fructose corn syrup (Hsu et al., 2015).

#### Neurotrophic Factors

Brain-derived neurotrophic factor (BDNF) is expressed abundantly in the hippocampus, where it plays an important role in neuronal growth, maintenance, and survival (Barde et al., 1982). Reductions in hippocampal BDNF have been proposed to underlie impairments in hippocampal-dependent forms of learning and memory (Leuner et al., 2006). BDNF is reduced in rats maintained on a high-fat, high-sugar diet and this reduction is accompanied by deficits in both spatial and nonspatial memory (Molteni et al., 2002; Kanoski and Davidson, 2011). Regarding spatial memory, Molteni et al. (2002) found reduced BDNF in the hippocampus, but not in the cerebral cortex of rats, after 2, 6, and 24 months on a Western-style diet. When these rats were tested in the water maze, lower levels of BDNF in the hippocampus were associated with worse spatial memory performance. Kanoski et al. (2007) reported that rats fed a high-fat, high-sugar diet for more than 90 days showed reduced levels of BDNF in both the ventral hippocampus and medial prefrontal cortex (mPFC), whereas BDNF levels in the dorsal hippocampus did not differ significantly from chow-fed controls. Neither the WD-fed or chow fed rats were impaired in learning a simple discrimination (e.g., tone signaled sucrose reward, light signaled no reward). However, the low-BDNF rats fed WD were significantly impaired when required to learn that the original discriminative contingencies had been reversed. Rats with hippocampal or mPFC damage are also impaired relative to intact controls in discrimination-reversal, but not in the original acquisition of a simple discrimination problem (Berger and Orr, 1983; Davidson and Jarrard, 2004).

#### Neuroendocrine Signaling

The hippocampus is densely populated with receptors for many neuroendocrine signals that contribute to the regulation of energy intake and body weight (for review see McGregor et al., 2015). Many of these signals have also been shown to influence learning and memory functions that depend on the hippocampus (for review see Kanoski and Grill, 2017). In addition, the operation of these signaling systems is susceptible to disruption by intake of WD. The particular neuroendocrine signals that are reviewed next appear to be involved with both energy regulation and cognition.

##### Insulin

This hormone, which is produced in pancreatic beta cells, is best known for its critical involvement in peripheral glucoregulation. Insulin also has important effects in the central nervous system. When infused directly into the brain, insulin decreases food intake and body weight (Woods and D’Alessio, 2008) and enhances hippocampal-dependent learning and memory (Park et al., 2000). Memory in humans is also enhanced when insulin is administered intravenously (Craft et al., 2003) or intranasally (Reger et al., 2008). In contrast, insulin resistance is associated with impairments on hippocampal-dependent behavioral tasks in rats (Fadel and Reagan, 2016) and in episodic memory in humans (Cheke et al., 2017). Other work shows that people with Type II diabetes, which is characterized by impaired insulin signaling, have double the risk of developing dementia (Ronnemaa et al., 2008; Cowie et al., 2009; Alford et al., 2018). Research with rodent models indicates that insulin transport into the brain may be reduced following intake of WD, thereby reducing the ability of insulin to enhance forms of memory that rely on the hippocampus Gray et al. (2017). Further, insulin resistance in the brain has been reported to develop after obesity, a finding that questions the direction of the causal relationship between these two disorders (Clegg et al., 2011).

##### Leptin

Released from fat cells, leptin can have dramatic suppressive effects on food intake and body weight regulation in both humans and rodents. In the hippocampus, leptin promotes both synaptic plasticity (Shanley et al., 2001) as well as neurogenesis (Garza et al., 2008). Furthermore, leptin signaling in the ventral hippocampus has been reported to interfere with the consolidation of memories for food locations (Kanoski et al., 2011). This effect of leptin signaling may actively inhibit processing of or attention to food-related features of the environment in favor of nonfood features, thereby reducing the likelihood that those features will evoke appetitive and eating behavior. Like insulin, leptin’s signaling capacity in the brain is reduced in conjunction with obesity. One mechanism to account for this type of brain “leptin resistance” is that leptin uptake at the BBB is reduced for subjects with higher levels of adiposity (Banks et al., 2004). If leptin has less access to the brain, it can exert less of an effect on the hippocampus. It is also possible that leptin resistance in the brain takes place at the cellular or receptor level, making leptin less effective, rather than less available (Munzberg et al., 2004). Whether the effects of WD consumption on leptin sensitivity or availability occur as a cause or an effect of obesity also remains to be elucidated.

##### GLP-1

Glucagon-like peptide-1 (GLP-1) is secreted from cells in the intestine and from neurons in the nucleus tractus solitarius (NTS). GLP-1 improves glucose homeostasis and decreases appetite (for review, see Steinert et al., 2017). GLP-1 degrades rapidly, but long-acting GLP-1 agonists, such as liraglutide, have been associated with decreased intake (Scott and Moran, 2007) and weight loss (Niswender et al., 2013). Other reports show that liraglutide reduces hippocampal neuroinflammation induced by fatty-acid exposure (Barreto-Vianna et al., 2017). While the prevailing hypothesis has been that GLP-1 suppresses appetitive responding *via* reducing food motivation or the incentive value of food (e.g., Dickson et al., 2012; Hayes and Schmidt, 2016), recent data suggest a role for GLP-1 in higher-order cognition. For example, Hsu et al. (2018a) identified a GLP-1 expressing, monosynaptic, glutamatergic pathway linking the ventral hippocampus to the medial prefrontal cortex (mPFC) that, when activated, is able to suppress food intake and improve impulse control in response to food cues. Jones et al. (2018b) compared the effect of GLP-1 on learned inhibition relative to reward value. While chronic administration of a liraglutide enhanced the ability of a learned inhibitory stimulus to suppress responding to a cue associated with food, it had no effect on responding when that food cue was presented alone. These data favor the hypothesis that GLP-1 suppresses appetitive behavior by enhancing learned inhibition rather than decreasing the rewarding or motivational properties of food.

##### Ghrelin

Unlike insulin, leptin and ghrelin increase eating and appetitive behavior (Cummings et al., 2001). These effects of ghrelin may involve the operation of memory processes (for review see Hsu et al., 2016). Diano et al. (2006) showed that ghrelin promoted neural plasticity in the hippocampus and improved memory consolidation in a hippocampal-dependent contextual passive avoidance task that required rats to remember that a specific place is associated with foot shock. Ghrelin delivered directly into the ventral hippocampus also stimulates meal initiation in free-feeding rats in response to stimuli (e.g., auditory tones) that previously signaled food availability when rats were food deprived (Kanoski et al., 2013). Thus, it may be that ghrelin in the ventral hippocampus modulates the ability of food-relevant cues to consolidate or retrieve the memory of reward produced by consuming a particular food. Furthermore, hippocampal ghrelin receptor activation modulates appropriate social transmission of food preference (Hsu et al., 2018b), providing another possible avenue for ghrelin to regulate intake. Activation of a ghrelin-positive, direct pathway between the hippocampus and the arcuate hypothalamic nucleus and medial amygdaloid nucleus may contribute to intake modulation by hippocampal, ghrelin-positive receptors (Russo et al., 2017). Kanoski et al. (2013) provided evidence that the hippocampus of rats maintained on WD becomes ghrelin resistant. However, the degree to which this diminished impact of ghrelin on feeding is associated with a reduced effect on hippocampal-dependent learning and memory function remains to be investigated. Also, it is not yet known whether ghrelin resistance in the hippocampus can be produced by WD intake independent of the effect of that diet on body weight and adiposity.

## Implications

It is abundantly clear that cognitive processes involved with attention, expectancy, memory, and inhibition are critically involved with the control of food intake and body weight. Interference with these processes may lead to overeating and body weight gain. This interference may result from environmental conditions that alter our attention to food and food-related events, weaken our ability to anticipate the consequences of eating, and disrupt memory mechanisms that help us to inhibit the tendency to eat. However, interference may also be a consequence of emergent pathophysiologies in brain substrates that underlie these cognitive functions. Moreover, many of the neurohormonal signals that have been identified as critically involved with energy homeostasis are known to have receptors in the hippocampus and to influence hippocampal-dependent learning and memory functions. A complete account of the cognitive control of eating and body weight will need to better elucidate how the actions of neurohormones, dietary factors, environmental food-related stimuli are interrelated.

**Figure 1:**
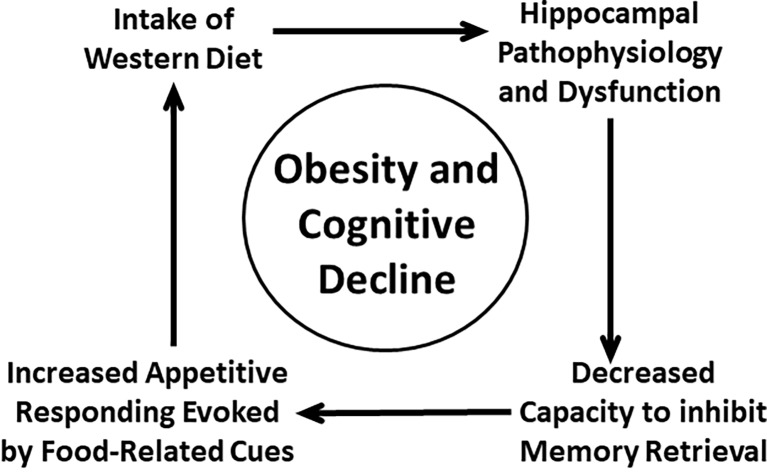
Vicious-cycle model of obesity and cognitive decline (adapted from Davidson, et al. 2005, 2007, 2014a,b; Kanoski and Davidson, 2011).

Another implication of the present review is that there may be a bi-directional relationship between cognitive dysfunction and energy intake dysregulation. Specifically, there is accumulating evidence that obesity and consumption of an obesity-promoting diet can lead to impaired brain functioning underlying cognition, especially in the hippocampus, and that impairment in hippocampal functioning can result in weakened cognitive control of food intake. We have attempted to resolve this apparent entanglement of cause and effect by positing that both energy dysregulation leading to obesity and cognitive dysfunction resulting from hippocampal pathophysiologies are part of a “vicious cycle” (Figure 1) where intake of diets high in sugar and saturated fats (e.g., Western diet) produces hippocampal dysfunction leading to weakened cognitive control of intake, which results in increased intake of harmful foods and beverages. The consequences of reiterations of this cycle can be obesity and cognitive decline (Davidson et al., 2005, 2007, 2014a; Kanoski and Davidson, 2011).

To fully comprehend the vicious-cycle model, a more detailed specification of some of the functions of the hippocampus will be useful. A number of findings agree with the view that the hippocampus is involved with the resolution of conflicts produced when a single event is associated with both rewarding and nonrewarding outcomes (Schumacher et al., 2016; White and Naeem, 2017). Although the formation of these competing associations does not depend on the hippocampus, the capacity of the inhibitory association to weaken retrieval of the memory of reward by antagonizing the excitatory association is hippocampal-dependent. This hippocampal function is based on its role in the utilization of contextual information. In brief, an ambiguous cue will excite the memory of reward, unless contextual cues signal that reward will not be forthcoming. That contextual information activates the inhibitory association, making the excitatory association less effective. To the extent that retrieval of the memory of postingestive reward is reduced, the ability of that memory to evoke appetitive behavior will also be diminished.

**Figure 2:**
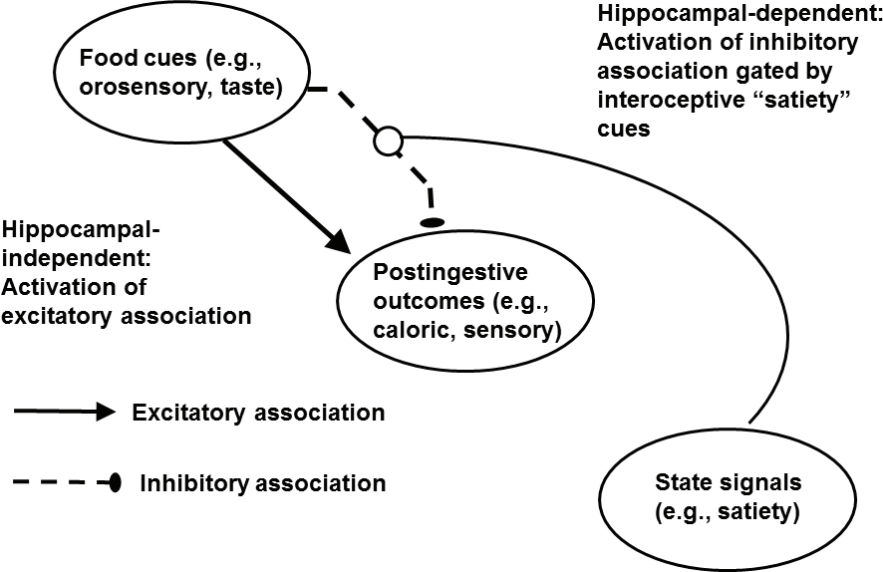
Mechanism underlying the learned control of intake by external food cues, memories of postingestive outcomes, and energy state signals. Adapted from Davidson et al., 2014a,b.

Momentarily setting aside the hippocampus, the associative mechanisms represented in the framework described here were first outlined by Bouton (1994) to explain the emergence of behavioral extinction (resulting from the reinforcement, then nonreinforcement of the same event) and counterconditioning (e.g., resulting from appetitive, then aversive reinforcement of the same event). Figure 2 shows how this framework applies to the cognitive control of energy regulation (Davidson et al., 2014a,b). We proposed that food and food-related stimuli in the environment are associated with positive postingestive outcomes on some occasions (e.g., before a meal) and are associated with postingestive consequences that are nonrewarding or even aversive under other circumstances (e.g., at the end of meal). The decision to eat or to refrain from eating depends on which of these conflicting associations is most strongly activated. To resolve this conflict, animals including humans can use at least two types of contextual information. First, interoceptive satiety signals gate or activate the inhibitory association by informing the animal that postingestive reward is not forthcoming. This activation makes it more difficult for the excitatory association to retrieve the memory of the rewarding postingestive stimulation produced by eating. The result is that the tendency to engage in appetitive behavior is reduced. Second, as demonstrated by Higgs (2008), the memory of a recently consumed meal can reduce subsequent intake. Because that recent memory can also serve as signal that food cues will not be followed by postingestive consequences for up to a few hours later, those memories can also serve as contextual cues that inhibit appetitive behavior by activating the inhibitory association (Davidson et al., 2014a; Higgs, 2016). From this perspective, impairments in episodic memory may promote excess intake by reducing the power of those memories to signal that food intake will not be postingestively rewarding. Moreover, as this mechanism is based on the encoding of relationships among contextual cues, food cues, and postingestive stimulation, it may operate in relational memory (see section Cognitive Impairments Earlier in Life). As noted previously, relational memory is impaired in obese children and those who consume high amounts of dietary fat. Relational memory has also been described as hippocampal-dependent (e.g., Khan et al., 2015). Thus, the associative framework outlined here may explain how hippocampal damage produced by surgical interventions and hippocampal pathophysiologies produced by consumption of a WD can severely reduce the ability of interoceptive satiety cues, memories, and other signals for the nonreinforcement of food cues, to gain inhibitory control of appetitive behavior.

The mechanism depicted in Figure 2 is derived primarily from research on rodents. However, an interesting translation of the main concepts shown in that diagram has been provided by Attuquayefio et al. (2016). These researchers found that for college students, eating food to satiety had different effects on the ratings of food liking (e.g., assessment of its palatability when eating it) relative to food wanting, dependent on their level of self-reported consumption of a high-fat, high-sugar (HFS) diet. Specifically, while a shift from hunger to satiety was associated with reductions in both liking and wanting, much smaller reductions in wanting were associated with higher levels of HFS intake. The authors proposed that while liking depends on an assessment of a food’s sensory consequences or palatability at the time it is consumed, wanting depends much more on one’s memory of prior experiences eating a given food at particular time, place, or set of conditions. Accordingly, just as WD is hypothesized to reduce the ability of satiety cues to antagonize the memory of the postingestive consequences of intake in the model shown in Figure 2, Attuquayefio et al. (also see Stevenson and Francis, 2017) proposed that high HFS intake by humans reduced the ability of satiety to inhibit retrieval of memories that evoked wanting. Higher intake of HFS was also predictive of weaker performance on a hippocampal-dependent paired-associate learning task. This inverse correlation was consistent with the hypothesis that an impairment in hippocampal functioning also underlies the weakened ability of satiety cues to suppress wanting.

The emphasis of our analysis on the hippocampus may seem neglectful of cognitive and sensory processes that are mediated by other brain substrates. A recent paper (Chen et al., 2018) presents a model that summarizes some of these hypothetically hippocampal-dependent processes but within a framework that we think agrees with some of the main features of our hippocampal-dependent associative perspective, and thus presents a potentially useful opportunity for integration. The Chen et al. model proposes that overeating and obesity are a consequence of an imbalance among three neurocognitive systems: (1) an insular-cortex-mediated “interoceptive awareness system” that translates homeostatic and interoceptive signals, presumably including satiation/hunger states, into awareness of desires/urges/cravings; (2) an “impulsive system,” which relies on the striatal-amygdala circuit to promote habitual, incentive-motivational responses to food and other rewards when triggered by food-related environmental cues; and (3) a “reflective” and inhibitory control system that resides primarily in the prefrontal cortex (PFC) and suppresses the tendency to engage in appetitive behavior.

Within this “tripartite model,” overeating leading to obesity is viewed as a consequence of a deficit in decision making that arises from an imbalance in these three systems. Specifically, the inability to resist tempting, typically energy dense, highly palatable food, results when food-related stimuli strongly evoke habitual appetitive and consummatory behavior (involving activation of the impulsive system), under conditions in which the ability to either inhibit the evocation of those responses and/or anticipate their negative consequences (involving the reflective system) is degraded. The imbalance is exacerbated by alterations in the interoceptive awareness system that potentiate the response-evoking power of the impulsive system while simultaneously weakening the reflective system’s capacity to exert inhibitory control over energy intake.

We agree that these three systems and their respective neural substrates are likely to have significant roles in the regulation of eating behavior and body weight. For example, similar to the impulsive system, we have suggested that the evocation of appetitive responses depends on the excitement of simple associations between food cues and rewarding postingestive outcomes. A striatal-amygdala circuit is a plausible substrate for this type of associative activation. Similar to the function of the reflective system, we proposed that suppression of appetitive behavior depends on the activation of an inhibitory association that antagonizes what may be the striatal-amygdala-dependent excitatory association between food cues and rewarding postingestive outcomes. It is likely that deciding to eat or not to eat involves inhibitory processes that depend on the PFC. However, we would also stipulate that decision making presumably depends, in part, on memories of past experiences, and we suggest that the tendering of this information for use by the PFC may depend on the hippocampus. Finally, similar to the interoceptive awareness system, we also assume that homeostatic signals arising from the internal milieu are a key source of information used to maintain energy balance.

In our view, the hippocampus is critically involved with utilizing that information. Thus, unlike the framework outlined by Chen et al. (2018), we see the coordinated operation of their three types of systems to be highly dependent on the hippocampus. This perspective is based on a wide variety of findings. In humans, direct reciprocal connections between the anterior hippocampus (corresponding to the ventral hippocampus in rats) and the amygdala, insula, and PFC make the hippocampus well-situated neuroanatomically to serve as an interface for all three regions (Poppenk et al., 2013). Moreover, our model describes how findings from studies aimed at specifying involvement of the hippocampus in learning and memory have identified functions (e.g., utilization of contextual cues, memory inhibition) that appear to have a significant role in the cognitive control of intake. Indeed, it would be surprising if the hippocampus was left out of this type of control. Perhaps the key function of the hippocampus in modulating eating an appetitive behavior is the utilization of interoceptive energy state information. Research with both rats and humans indicates that interference with hippocampal functioning produced by either surgical ablation or more selective neurotoxic interventions (e.g., Hebben et al., 1985; Davidson and Jarrard, 1993; Kennedy and Shapiro, 2004; Davidson et al., 2010) or by intake of high-energy Western-style diets (Francis and Stevenson, 2011; Sample et al., 2018) impairs the ability to discriminate between interoceptive energy state cues corresponding to hunger and satiety. As described in our model and elsewhere (Kennedy and Shapiro, 2004, 2009; Boutelle and Bouton, 2015; Schepers and Bouton, 2017), interoceptive energy state cues corresponding to hunger and satiety can be characterized as contextual stimuli which modulate the strength of the predictive relationships between food-related stimuli and their postingestive outcomes. Memories of past experiences with these relationships would be important determinants of one’s expectancies about the outcomes of current and future encounters with these cues. This information, forwarded from the hippocampus to the PFC, potentially involving recently established signaling pathways (Hsu et al., 2018a), could then be used in decision making related to the inhibitory control of intake. Accordingly, impairments in the detection of interoceptive satiety cues, or in the use of the contextual information they provide, would reduce an important counter to the evocation of eating appetitive behavior by environmental stimuli. This could be part of the mechanism that underlies overeating and body weight gain.

## Questions and Future Directions

There are many open questions associated with the above analysis that merit investigation. As noted by Yeomans (2017), evidence for the vicious-cycle model and the effects of WD on hippocampal-dependent cognitive functioning has been derived primarily from research on rodents and thus translational aspects of the model need to be addressed. As noted above, some recent findings from human studies (e.g., Attuquayefio et al., 2016; also see Stevenson and Francis, 2017) are consistent with the interpretation of the rodent experiments. Although much previous research has examined the effects of obesity on human cognition, additional studies are needed to assess the effects of diet and WD specifically on human expectancy, memory, perception, attention, and inhibitory processes which underlie energy regulation.

Consistent with available data, the vicious-cycle model suggests that even relatively short-term exposure to WD can have harmful effects on hippocampal-dependent cognitive functioning which may develop into more serious cognitive impairments as a consequence of long-term exposure. In fact, some reports indicate that animals recover from cognitive deficits and some signs of pathophysiology (reduced glucose transport) that are observed following initial WD exposure but that cognitive impairment reappears along with new pathophysiologies (e.g., increased BBB permeability) following continued exposure to WD (e.g., Hargrave et al., 2016; Jais et al., 2016). The mechanisms that underlie the short-term and long-term effects of WD, the processes that underlie recovery following short-term WD exposure, and whether such recovery occurs at all in humans remain to be investigated. For example, it could be that mechanisms involved with initial exposure to WD are subject to recovery, whereas potentially different processes activated as a consequence of more delayed and gradually increasing adiposity are not (see Wang et al., 2015).

Perhaps the most fundamental question generated by the vicious-cycle model is how to break the cycle. An obvious answer is to restrict intake of food high in fat and sugar. Unfortunately, perhaps the most pernicious aspect of the vicious cycle is that the ability to control one’s own intake is curtailed as a consequence of WD exposure. This suggests that research aimed at protecting the brain and the hippocampus specifically from the harmful effect of WD may be most fruitful. This could include further research to evaluate and improve the effects of inhibitory control training as well as research on developing interventions directly aimed at reducing diet-induced hippocampal pathophysiologies. In this regard, studies of GLP-1 analogs, which appear to act as endogenous satiety signals and may also enhance cognitive inhibitory processes (Jones et al., 2018a) as part of a ventral hippocampal-prefrontocortical signaling pathway (Hsu et al., 2018a,b), have promise. More generally, the development of interventions would benefit greatly by research that improves understanding of the mechanisms underlying WD- or obesity-induced hippocampal pathophysiologies (e.g., BBB disruption, interference with glucose transport, inflammation, reduced BDNF) and whether they emerge independently or based on common underlying mechanisms.

## Concluding Remarks

It is clear that memory, expectancy, inhibitory, and decision-making processes are important contributors to the control of eating and body weight. Furthermore, accumulating evidence indicates that the hippocampus may be involved with each of these types of cognitive controls. Interference with hippocampal function, produced by lesions or temporary inactivation, is followed by impaired control of appetitive behavior including shortened intermeal intervals, increased intake, body weight gain, and a reduced ability to discriminate between interoceptive stimuli related to energy state. However, there is little evidence that recent dramatic worldwide increases in the incidence of overweight and obesity are attributable to lesioned or inactivated hippocampi. This makes findings that exposure to obesity-promoting high-energy diets (e.g., Western diets) recapitulate many of the effects on feeding behavior, energy, and body regulation that are produced by more invasive hippocampal manipulations, especially noteworthy. These dietary effects are accompanied by signs of hippocampal pathophysiology, such as inflammation, reduced BDNF levels, reduced expression of glucose transporters, and selective increases in permeability of the hippocampal BBB. Moreover, these diets have also been shown to impair well-established hippocampal-dependent learning and memory processes in both human and nonhuman animals. The available data are consistent with a vicious-cycle model which proposes that Western diet intake produces pathophysiologies that interfere with the hippocampal-dependent ability to antagonize the retrieval of food reward memories by environmental food-related stimuli, thereby promoting additional consumption of Western diet. Some of the hippocampal pathophysiologies associated with Western diet consumption are also characteristic of Alzheimer’s disease and Alzheimer’s-like dementias. Accordingly, future research on the vicious-cycle model should investigate its implications not only for obesity and metabolic disorders, but also for the emergence of serious forms of late-life cognitive decline.

In many ways, this review highlights the continued blurring of the distinction between what have been labeled “homeostatic” (i.e., physiological) and “nonhomeostatic” (i.e., learned) controls of eating. Physiological processes can be seen as providing critical information (e.g., neurohormonal satiety signals; postingestive stimuli) that is encoded, remembered, retrieved, and translated into relationships that guide appetitive and eating behavior. In fact, it may be that the power of satiation to suppress intake depends on the success of this translation or on the ability to use it to predict the consequences of eating. Furthermore, from this perspective, the brain substrates of cognition can be viewed as no less important for maintaining energy balance than are other forebrain and hindbrain sites that have been viewed more traditionally as the sites of homeostatic control. Similarly, interference with neurohormonal processes that help regulate intake may contribute to cognitive decline. Research aimed at describing how cognitive and noncognitive control processes and substrates are integrated has much promise for improving understanding of energy regulation, dysregulation, as well as diet and obesity-related challenges to cognitive functioning.

## Author Contributions

All authors contributed to the writing of the manuscript. TD was primarily responsible for the conception of the work and its organization content. SJ and RS contributed, additional content, organization, and editing. MR contributed to the editing and literature search and interpretation of the products of that search.

### Conflict of Interest Statement

The authors declare that the research was conducted in the absence of any commercial or financial relationships that could be construed as a potential conflict of interest.
